# Recurrent Anterior Shoulder Instability Treated Using the Arthroscopic Bankart–Latarjet Technique: Experience of a Peripheral Hospital

**DOI:** 10.3390/jcm12165274

**Published:** 2023-08-14

**Authors:** Alban Fouasson-Chailloux, Daniel Estoppey, Alan Perdreau, Charles Bessière, Yariv Goldstein, Christophe Duysens

**Affiliations:** 1 Institut Européen de la Main, Hôpital Kirchberg, 9 Rue Edward Steichen, L-2540 Luxembourg, Luxembourg; daniel.estoppey@gmail.com (D.E.); duysensc@gmail.com (C.D.); 2 Médecine Physique et Réadaptation Locomotrice, CHU Nantes, Nantes Université, 44093 Nantes, France; 3 Inserm, UMR 1229, RMeS, Regenerative Medicine and Skeleton, ONIRIS, Nantes Université, 44042 Nantes, France; 4 Service D’orthopédie, Vivalia, Centre Hospitalier de l’Ardenne, Avenue de Houffalize 35, 6800 Libramont-Chevigny, Belgium; alan.perdreau@vivalia.be; 5 OrthoVar, Pôle Médical Epsilon 3, 87 Avenue Archimede, 83700 Saint Raphaël, France; charles.bessiere@gmail.com; 6 Assuta Samsom University Hospital, 7 HaRefu’ah St., Ashdod 747629, Israel; yariv.goldstein@gmail.com

**Keywords:** shoulder instability, arthroscopic, complications, peripheral hospital center, clinical scores

## Abstract

The arthroscopic Bankart–Latarjet procedure is used in the surgical management of anterior shoulder instability. This technique is mainly performed in referral centers due to its high technicity. This study aimed to evaluate surgical outcomes in a peripheral hospital center. This is a retrospective study of patients treated for recurrent anterior shoulder instability. The clinical scores (Walch–Duplay, Rowe, and Western Ontario Shoulder Instability Index (WOSI)) were assessed preoperatively and at 12 months after surgery. The consolidation and the position of the bone block were evaluated at 6 months using a CT scan. Between 2016 and 2020, 40 patients had been operated on (mean age: 28.5 ± 7.9 years). During a mean follow-up of 29.5 ± 11.6 months, we noted only one complication, a case of fracture of the callus of a consolidated bone block. No recurrence of instability was recorded. The Walch–Duplay score increased from 17.8 to 94.6, the Rowe score from 24.9 to 96.8, and the WOSI score decreased from 52.1% to 6.9%. The bone block was consolidated in 35 patients (87.5%), and a flush position with the anterior edge of the glenoid was noted for all patients. At one year, 67.0% of the patients practicing sport had returned to sports. The arthroscopic Bankart–Latarjet technique was a reliable procedure in the hands of an experienced shoulder surgeon, even in a peripheral hospital center.

## 1. Introduction

Contact sports in young athletic populations are responsible for high rates of shoulder injuries, especially shoulder dislocations [1]. For example, in the U.S.A., during the 2015–2019 period, 89,511 athletic-related shoulder dislocations were reported across the country [2]. This injury particularly concerns male athletes in nearly 90% of cases [2]. Sports such as basketball, rugby, and American football are particularly risky [1,3,4]. Shoulder dislocation may lead to recurrent anterior joint instability responsible for time lost from play [3,4]. In the case of recurrent instability, surgical procedures seem necessary, as they allow good results with regards to returning to sport for contact athletes in 75 to 100% of cases [5,6], except for overhead athletes, who seem to show more difficulties in returning to play [6].

Coracoid transfer with screw fixation was described by Albert Latarjet in 1954 [7]. This technique has since remained the technique of choice for the treatment of recurrent anterior shoulder instability [8,9]. In 2007, Lafosse et al. described the first all-arthroscopic Latarjet procedure with the use of two cannulated screws [10]. In an effort to address the specific complications related to screw fixation of the bone block [11], Boileau et al. developed the arthroscopic Bankart–Latarjet procedure, a system using cortical suture buttons for fixation of the bone block [12]. This technique preserves and repairs the anterior labrum. Restoring the labrum, in addition to bone block repair, improves stability and proprioception, as it may have a long-term protective effect against osteoarthritis [13,14]. However, due to its high technicity, arthroscopic Bankart–Latarjet surgery is mainly performed in large referral centers that operate on a large number of patients. Performing such a procedure in smaller or peripheral hospitals with a lower number of patients may be disputable.

This study aimed to evaluate the surgical outcomes of the arthroscopic Bankart–Latarjet procedure in a peripheral hospital (10 to 15 procedures a year) by an experienced shoulder surgeon (experience level III according to Tang et al. [15]). Specifically, the study focused on short- and mid-term complication rates, clinical and functional scores, and radiological results (position and consolidation of the bone block).

## 2. Materials and Methods

### 2.1. Population

This retrospective study assessed patients operated on between September 2016 and February 2020 for recurrent anterior instability of the shoulder. Primary surgeries or revision surgeries after the failure of an isolated arthroscopic Bankart were included. The inclusion criteria were a glenoid bone loss ≥ 40% according to the Gerber index [16] and a preoperative osteoarthritis stage ≤ I according to Samilson–Prieto [17]. The preoperative patient assessment was performed by the surgeon and included:

- A measure of shoulder range of motion (ROM) in degrees: forward flexion, external rotation with the elbow tucked into the body (ER1) and at 90° of abduction (ER2), and internal rotation (IR1) with the hand behind the back (thumb to D6, D12, L3, or S1 vertebra);
- A CT-arthrography of the shoulder;
- A record of Walch–Duplay, Rowe, and Western Ontario Shoulder Instability Index (WOSI) scores [18,19,20]. Briefly, the Walch–Duplay score is a four-item clinical score assessing shoulder activity, stability, pain, and mobility and is interpreted as follows: excellent (from 91 to 100 points), good (76 to 90 points), fair (51 to 75 points), or poor (under 50) [18]. The Rowe score assesses three items: stability on 50 points, mobility on 20 points, and function on 30 points. The score is considered excellent from 90 to 100 points, good between 89 and 75, fair between 74 and 51, and poor below 50 [20,21]. The WOSI is a 21-item self-administered questionnaire focusing on quality of life related to shoulder instability from 0 (best score) to 2100 (worst score) [19,22].

The analysis of the data was carried out after anonymization. The ethics committee, Comité Nantais d’Ethique en Médecine du Sport, under ethical committee registration CNEMS-2023-001, approved the study, which was in accordance with the declaration of Helsinki [23].

### 2.2. Surgical Procedure

All of the procedures were performed by the same orthopedic surgeon with 12 years of experience who performs about 120 shoulder surgeries annually. He had the assistance of a scrub nurse and, occasionally, an assistant, with the patient under general anesthesia and interscalene block. The patient was positioned in the beach chair position (30–40°) with the ipsilateral limb placed on a movable arm positioner (Spider Limb Positioner, Smith & Nephew, Andover, MA, USA). Systolic blood pressure was, when possible, kept below 100 mmHg. The surgical technique used specifically designed equipment for the arthroscopic surgical procedure (Latarjet Guiding System; Smith & Nephew Inc., Andover, MA, USA) and was previously described by Boileau et al. [12]. Briefly, the technique followed five steps: (1) coracoid preparation, drilling, and osteotomy; (2) glenoid preparation and drilling; (3) subscapularis splitting and axillary nerve protection; (4) coracoid transfer and fixation; and (5) Bankart repair.

The operating time (from incision to skin closure) was recorded. The following intraoperative events were noted: conversion to open surgery, the occurrence of a fracture of the bone block, or an occurrence of a hardware-related complication.

### 2.3. Postoperative Procedure and Follow-Up

Postoperatively, the shoulder was immobilized in a neutral rotational splint day and night for 3 weeks and then only at night for 3 weeks. Carrying a load superior to 1 kg was prohibited for 6 weeks in concentric mode and for 3 months in eccentric mode. Self-rehabilitation via pendular swinging and active-assisted movements began in the first week after surgery. Active movements and pool exercises started on the 7th week after surgery, and muscle strengthening was not allowed before the 13th week. Return to sport was allowed between 3 and 6 months after surgery.

Patients were clinically examined by the surgeon at 2, 6, and 12 weeks and then at 6 and 12 months postoperatively. The following parameters were noted: the presence of a hematoma; infection of the surgical site; neurological injury; recurrent subluxation or dislocation; detachment or laxity of the cortical suture buttons; and need for surgical revision.

Postoperative incidents were defined as “problems” if they did not change the prognosis; as “complications” if they required surgical revision or negatively influenced the prognosis; and as “trauma” if they were related to a new traumatic event [24]. At 12 months after surgery, joint mobility, clinical scores, and apprehension tests were performed.

**Figure 1:**
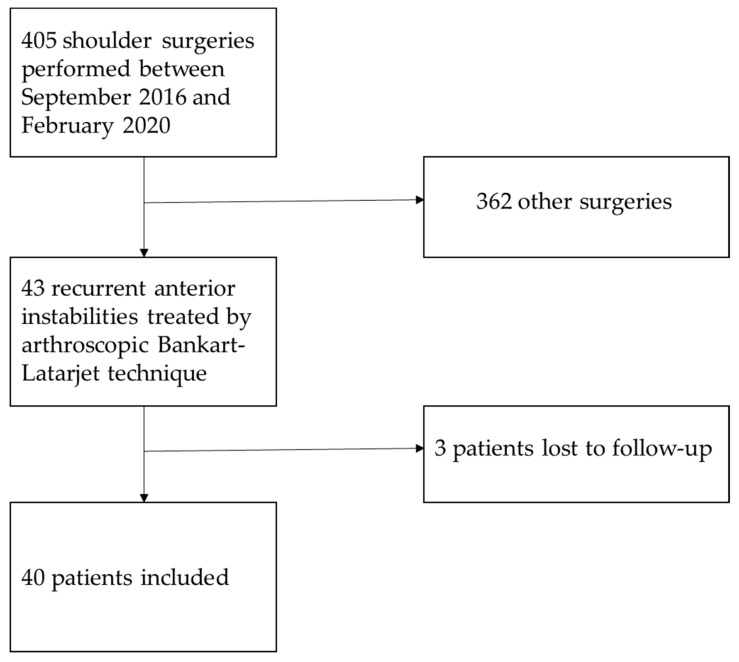
Flow diagram of the study.

The position and the union of the bone block were evaluated using a CT scan at 6 months after surgery (Figure 1) and, if needed, another CT scan at 12 months. They were analyzed by the surgeon and by an independent orthopedic surgeon. Inter-observer reliability of shoulder assessment via CT scan has previously been described from good to excellent (intra-class correlation coefficients from 0.735 to 0.968) [25]. The position of the block was considered “ideal” when it was located below the “equator line” (a line perpendicular to the intersection of the line joining the supra- and infra-glenoid tubercles at the medial height of the glenoid) and flush with the anterior edge of the glenoid [14]. It was considered equatorial or supra-equatorial if, respectively, more than 25% or more than 50% of the block was above the equator [12]. It was considered too lateral or too medial if its lateral edge was, respectively, protruding by more than 3 mm or located more than 5 mm medially from the anterior rim of the glenoid [26]. Pseudarthrosis was defined by a border of less than 5 mm on the CT scanner at one year. Beyond 5 mm, the block was considered migrated [14]. The occurrence or the worsening of osteoarthritis was noted.

### 2.4. Statistical Analysis

Continuous quantitative variables were expressed in mean and standard deviations, while qualitative variables were expressed as absolute values and percentages. Statistical analysis was carried out with JMP software (SAS Institute, Cary, NC, USA). Statistical significance was established for a *p*-value < 0.05. Univariate analysis between qualitative variables was performed by applying Fisher’s exact test. The comparative analysis of continuous quantitative variables between two independent groups was performed with the Mann–Whitney U test and with the Wilcoxon signed rank test in the case of paired data.

## 3. Results

### 3.1. Population

Forty-three operations were performed during the period of inclusion. Three patients were lost to follow-up and, therefore, excluded from the results (Figure 1). Forty patients were finally included (mean age: 28.5 ± 7.9 years). Thirty-six patients practiced sports before surgery (90%). The mean follow-up was 29.5 ± 11.6 months, ranging from 12 to 59 months. The patients’ demographics are summarized in Table 1.

### 3.2. Complications, Clinical and Radiographic Evaluation

The average operating time was 176 ± 34.4 min, ranging from 120 to 285 min. Five patients presented a concomitant SLAP lesion, which was treated at the same time. There was no need for conversion to open surgery, and we noted no bone block fractures or hardware-related complications. One patient presented an occipital pressure sore at the location of the headrest (probably related to an operating time of 285 min); this resolved spontaneously. Therefore, we defined the incident as a “problem”.

No postoperative complications were observed. No revision was needed. One patient presented a fracture of the bone block callus, which had previously been consolidated, following a motorcycle accident, one year after surgery. We defined the incident as “trauma”. This fracture was treated conservatively and was consolidated on the CT scan performed 6 months after the accident.

At 12 months after surgery, clinical scores improved significantly compared to the preoperative records (Table 2). Concerning shoulder ROM, forward flexion increased from 158.5° ± 15.6 to 168.5° ± 10.5 (*p* < 0.001), and ER2 increased from 80.6° ± 15.0 to 86.8° ± 17.3 (*p* = 0.03). ER1 and IR1 were unchanged (*p* = 0.27 and 0.12, respectively) (Table 2). Two patients presented a positive apprehension test that persisted at 12 months. Twenty-four of the patients practicing sports (67.0%) had returned to their previous sport at this time point.

In the case of a bone block procedure performed as a revision of a previous surgery, there was a significantly poorer WOSI score (Table 3). An age of less than 20 years at the time of surgery showed a favorable influence on the WOSI score at 12 months (1.2% vs. 7.5% (*p* < 0.03)). Work-related trauma or worker compensation did not significantly reduce the postoperative clinical scores.

Twenty-eight bone blocks (70.0%) were subequatorial, eleven (27.5%) equatorial, and one (2.5%) supraequatorial. The forty bone blocks (100%) were flush with the anterior margin of the glenoid. Thirty-five bone blocks (87.5%) consolidated; two (5.0%) were in pseudarthrosis, and three (7.5%) had migrated or were almost completely absorbed.

Even if a trend was noticed, smoking (23.0% vs. 7.4%, *p* = 0.31) and revision surgery (28.6% vs. 9.1%, *p* = 0.20) did not significantly increase the risk of non-union. Similarly, patients over 30 (25.0% vs. 4.2%, *p* = 0.13) and over 40 (50.0% vs. 8.3%, *p* = 0.07) had no more significant risk of non-union.

Non-union of the block had a significant negative impact on all clinical scores at 12 months (Table 4). On the contrary, the equatorial or supraequatorial positioning of the bone block did not significantly influence those scores. Two patients had pre-existing osteoarthritis, which evolved from stage I to II according to the Samilson–Prieto classification.

## 4. Discussion

This study confirmed that arthroscopic Bankart–Latarjet is a technically difficult procedure, as seen by the average operating time of 176 min. Other series of arthroscopic Latarjet procedures (using either cortical suture buttons or screws) performed by surgeons with level IV experience (according to the classification in Tang et al. [15]) in referral centers with a large volume of activity obtained average times ranging from 76 to 161 min [27,28,29,30,31,32,33,34,35,36]. Only selected surgeons (including level V) can perform this procedure within an hour [33,37].

This study also confirmed that the arthroscopic Bankart–Latarjet technique guided by specifically designed instrumentation and fixed with cortical suture buttons is safe, even when performed in a peripheral hospital center with few surgeries of this type per year. In fact, our rate of pre- and postoperative bone block fractures (apart from the postoperative “traumatic” case) was zero. This rate is similar to [12,28,30,38,39,40,41] or lower than (intraoperative fracture of 1–10%; postoperative fracture of 0.8–5.7%) other series [27,29,31,32,33,37,42,43,44,45,46]. The use of suture buttons instead of screws allowed a drill hole in the bone block of only 2 mm in diameter and controlled and progressive tensioning with the aid of a dynamometer during the positioning and the fixation. This probably helped to prevent bone block fractures [39].

Interestingly, no early complications, such as infections or bruises, were found in this series. However, these early complications can reach up to 10.5%, depending on the series [12,27,28,29,30,31,32,33,37,38,39,40,41,42,43,44,45,46]. The use of specifically designed instrumentation certainly protected the nerves (axillary, musculocutaneous, and suprascapular nerves) due to the reproducible viewing angle of the glenoid and the flexible guidance of the block until its positioning and final fixation with the aid of the dynamometer [12,27,39,41,42]. The use of screws as a means of fixation has conflicting results in the literature, depending on the series. It is sometimes considered safe [28,29,30,31,38,40,43] and sometimes risky (1–1.5%) [32,33,37,44,45,46].

None of our patients presented recurrent instability up to the end of the follow-up. Other series found similar [28,31,33,37,43] or higher (1.3 to 6.1%) rates [12,27,29,30,32,38,39,40,42,44,45,46] of recurrent instability. Moreover, no revision surgery was needed in this series using suture buttons. On the contrary, screws can lead to a revision rate of between 1 and 20.5% for hardware removal [29,31,32,33,37,38,40,43,44,45,46]. Fixation with suture buttons makes it possible to avoid this type of revision [12,27,39,42]. Generally, the overall revision rate after arthroscopic Latarjet ranges from 0% to 30.3%, depending on the series [12,27,28,29,30,31,32,33,37,38,39,40,41,42,43,44,45,46].

From a functional point of view, patients operated on with this technique had very good results, which were close to the results described in the literature. Indeed, at 12 months after surgery, the mean Walch–Duplay score of 94.6 was similar to other series (from 79 to 95.6 at the end of the follow-up); the mean Rowe score of 96.8 was close to other series (from 80 to 94 at the end of follow-up); and the mean WOSI score of 6.9% seemed better than in other series (from 9.4% to 21%) [12,27,28,29,30,31,38,39,40,41,43,45,46]. Surprisingly, we noted that non-union significantly reduced Rowe and WOSI scores, whereas Vadalà et al. showed the opposite for the open Latarjet [47]. We also reported a significant drop in the WOSI score in the cases performed as a revision after a previous Bankart surgery, unlike Dumont et al. [40]. Another favorable measurable outcome is the decrease in postoperative apprehension. Regarding our series, there was a low rate of persistent anterior apprehension (5% at 12 months after surgery, which is rather in the low range, compared to the other series, ranging from 0 to 50% [27,28,30,38,39,43,46]). This may be related to the addition of a capsular reconstruction (Bankart) to the coracoid bone block transfer [14]. We reported that 67% of the patients practicing sports before surgery had returned to their previous sport, which is a good result, considering that overhead athletes have a return rate of between 46% and 79% [6].

The union rate was 87.5%, which was comparable to other series using cortical suture buttons (82.8% to 100%) or screws (72.9% to 100%) [12,27,28,30,32,37,39,41,42,43,45,46,48]. Our optimal horizontal positioning rate of 100% exceeded those of series using cortical suture buttons (from 67% to 96%) and equaled those using screws (from 77% to 100%) [12,27,28,31,33,37,39,41,42,43,45,46,48]. The specially designed glenoid guide probably contributed to the flush position of the block [12]. The flexibility of the cortical suture buttons as well as the resorption of bone also allowed the correction of the position of bone blocks that were initially positioned too laterally [39,41].

On the other hand, we achieved what we considered to be an optimal position in the vertical plane in only 70% of the cases, which was lower than other surgical series using either cortical suture buttons (88% to 96%) or screw fixations (77.4% to 98%) [12,27,28,31,33,37,39,41,42,43,45,46,48]. In a cadaveric study, Lädermann et al. showed that the level of opening of the subscapularis using the “inside-out” technique (as used in our series) was rarely optimal. In fact, in 75% of cases, the split was in the upper third or at the junction between the upper and middle third of the subscapularis but never in the junction between the middle and lower third, as desired ideally [49]. This could, therefore, explain the difficulty in obtaining a correct position of the block in the vertical plane. Fortunately, this non-optimal position did not negatively influence the clinical scores and did not increase the rate of recurrence. Due to its assumed proprioceptive, stabilizing, and protective effect against osteoarthritis, capsular-labral repair may have contributed to these good results [13,14].

From a medical–economic point of view, the arthroscopic Latarjet is more than twice as expensive as the classic open technique. Indeed, Randelli et al. estimated, in their systematic review, an average direct cost (cost of the operating room and the equipment) per intervention of EUR 2335 for the arthroscopic technique with screws and EUR 1040 for the open technique [50]. Currently, no data exist comparing the indirect costs between the two techniques (loss of labor and salary; family and social costs; re-operations for the removal of hardware in particular).

Finally, this study has some limitations. The first one is the retrospective design of the study, without a control group, in a population that may appear small and heterogeneous. The second one is the rather short follow-up, which might underestimate some late complications. The third one could be the clinical follow-up performed by the patient’s surgeon, who also assessed the postoperative CT scans with an independent orthopedic surgeon and not with an independent radiologist. Nevertheless, this study has the advantage of reflecting the activity of a shoulder surgeon in a small peripheral hospital and can, therefore, serve as a model for surgeons practicing in small hospitals who may aim to perform the arthroscopic Bankart–Latarjet technique.

## 5. Conclusions

The Bankart–Latarjet arthroscopic surgical technique using specially designed instrumentation with cortical suture button fixation is a difficult but safe and effective procedure when performed by an experienced surgeon, even in a peripheral center with few procedures per year. Consequently, while this surgery is currently rather reserved for large referral centers, it could be extended to peripheral hospital centers. However, it requires surgeons well-trained in this technique.

## Figures and Tables

**Table 1:** Demographic characteristics of the included patients.

Mean age (years) ± SD [min–max]	28.5 ± 7.9 [16–46]
Mean Instability Severity Index Score ± SD [min–max]	4.4 ± 1.2 [3–7]
Sex, Male/Female, n (%)	36 (90.0%)/4 (10.0%)
Dominant arm, n (%)	22 (55.0%)
Right arm, n (%)	23 (57.5%)
Sport practice, n (%)	36 (90.0%)
Overhead or contact sport, n (%)	32 (80.0%)
Competitive athlete, n (%)	14 (35.0%)
Light manual worker, n (%)	4 (10.0%)
Heavy manual worker, n (%)	16 (40.0%)
Work-related trauma, n (%)	11 (27.5%)
Smoker, n (%)	13 (32.5%)
Failure of isolated arthroscopic Bankart, n (%)	7 (17.5%)
Hill–Sachs lesion, n (%)	36 (90.0%)
Concomitant SLAP lesion, n (%)	5 (12.5%)
Preoperative osteoarthritis *, n (%)	2 (5.0%)

Abbreviations: SD: standard deviation; Min: minimal value; Max: maximal value. * stage I according to Samilson–Prieto.

**Table 2:** Patients’ preoperative and 12-month postoperative shoulder range of motion and clinical scores (n = 40).

Clinical Outcomes	Preoperative	12 Months after Surgery	*p*
Forward flexion (°) ± SD [min–max]	158.5 ± 15.6 [150–180]	168.5 ± 10.5 [150–180]	<0.001
ER1 (°) ± SD [min–max]	74.8 ± 13.9 [40–90]	71.7 ± 16.9 [40–100]	0.27
ER2 (°) ± SD [min–max]	80.6 ± 15.0 [30–90]	86.8 ± 17.3 [30–120]	0.03
IR1:			0.12
D6, n	7	12	
D12, n	27	19	
L3, n	6	8	
S1, n	0	1	
Walch–Duplay ± SD [min–max]	17.8 ± 14.6 [0–50]	94.6 ± 9.4 [70–100]	<0.0001
Rowe ± SD [min–max]	24.9 ± 12.0 [0–50]	96.8 ± 6.8 [75–100]	<0.0001
WOSI, % ± SD [min–max]	52.1 ± 17.7 [15–80]	6.9 ± 8.7 [0.19–34]	<0.0001

Abbreviations: SD: standard deviation; Min: minimal value; Max: maximal value; ER1: external rotation with the elbow tucked into the body; ER2: external rotation with the elbow at 90° of abduction; IR1: internal rotation with the hand behind the back (thumb to D6, D12, L3, or S1 vertebra).

**Table 3:** Comparison of the patients’ clinical scores at 12 months after surgery between revision surgeries and primary surgeries.

Scores	Revision Surgery n = 7	Primary Surgery n = 33	*p*
Walch–Duplay ± SD	91.4 ± 12.1	95.3 ± 8.8	0.38
Rowe ± SD	95.0 ± 7.6	97.1 ± 6.6	0.33
WOSI (%) ± SD	12.4 ± 9.9	5.7 ± 8.1	<0.01

Abbreviations: SD: standard deviation.

**Table 4:** Comparison of the clinical scores at 12 months after surgery between patients with unconsolidated bone blocks and consolidated bone blocks.

Scores	Unconsolidated Bone Blocks n = 5	Consolidated Bone Blocks n = 35	*p*
Walch–Duplay ± SD	83.0 ± 11.0	96.3 ± 8.1	<0.01
Rowe ± SD	86.0 ± 9.6	98.2 ± 4.7	<0.01
WOSI (%) ± SD	19.8% ± 13.5	5.1% ± 6.1	0.02

Abbreviations: SD: standard deviation.

## Data Availability

The data presented in this study are available upon request from the corresponding author. The data are not publicly available due to ethical reasons.
